# *GATA2* zinc finger 1 mutations are associated with distinct clinico-biological features and outcomes different from *GATA2* zinc finger 2 mutations in adult acute myeloid leukemia

**DOI:** 10.1038/s41408-018-0123-2

**Published:** 2018-08-31

**Authors:** Feng-Ming Tien, Hsin-An Hou, Xavier Cheng-Hong Tsai, Jih-Luh Tang, Yu-Chiao Chiu, Chien-Yuan Chen, Yuan-Yeh Kuo, Mei-Hsuan Tseng, Yen-Ling Peng, Ming-Chih Liu, Chia-Wen Liu, Xiu-Wen Liao, Liang-In Lin, Chien-Ting Lin, Shang-Ju Wu, Bor-Sheng Ko, Szu-Chun Hsu, Shang-Yi Huang, Ming Yao, Wen-Chien Chou, Hwei-Fang Tien

**Affiliations:** 1 Division of Hematology, Department of Internal Medicine, National Taiwan University Hospital, Taipei, Taiwan; 2 Graduate Institute of Clinical Medicine, College of Medicine, National Taiwan University, Taipei, Taiwan; 3 Tai-Cheng Stem Cell Therapy Center, National Taiwan University, Taipei, Taiwan; 4 Greehey Children’s Cancer Research Institute, University of Texas Health Science Center at San Antonio, San Antonio, TX USA; 5 Graduate Institute of Oncology, College of Medicine, National Taiwan University, Taipei, Taiwan; 6 Department of Pathology, National Taiwan University Hospital, Taipei, Taiwan; 7 Department of Clinical Laboratory Sciences and Medical Biotechnology, College of Medicine, National Taiwan University, Taipei, Taiwan; 8 Department of Laboratory Medicine, National Taiwan University Hospital, Taipei, Taiwan

**Keywords:** Acute myeloid leukaemia, Cancer genetics

## Abstract

Mutations of the GATA binding protein 2 (*GATA2*) gene in myeloid malignancies usually cluster in the zinc finger 1 (ZF1) and the ZF2 domains. Mutations in different locations of *GATA2* may have distinct impact on clinico-biological features and outcomes in AML patients, but little is known in this aspect. In this study, we explored *GATA2* mutations in 693 *de novo* non-M3 AML patients and identified 44 *GATA2* mutations in 43 (6.2%) patients, including 31 in ZF1, 10 in ZF2, and three outside the two domains. Different from *GATA2* ZF2 mutations, ZF1 mutations were closely associated with French-American-British (FAB) M1 subtype, *CEBPA* double mutations (*CEBPA*^double-mut^), but inversely correlated with FAB M4 subtype, *NPM1* mutations, and *FLT3*-ITD. ZF1-mutated AML patients had a significantly longer overall survival (OS) than *GATA2-*wild patients and ZF2-mutated patients in total cohort as well as in those with intermediate-risk cytogenetics and normal karyotype. ZF1 mutations also predicted better disease-free survival and a trend of better OS in *CEBPA*^double-mut^ patients. Sequential analysis showed *GATA2* mutations could be acquired at relapse. In conclusion, *GATA2* ZF1 mutations are associated with distinct clinico-biological features and predict better prognosis, different from ZF2 mutations, in AML patients.

## Introduction

GATA binding protein 2 (GATA2) belongs to the GATA family of transcription factors which regulate hematopoietic stem cell proliferation and differentiation^1,2^. *GATA2* mutations have been reported in acute myeloid transformation of chronic myeloid leukemia (CML)^3^, familial myelodysplastic syndrome-related acute myeloid leukemia (MDS/AML), pediatric MDS^4,5^, Emberger syndrome^6^, and monocytopenia and mycobacterial infection (MonoMAC) syndrome^7,8^. Mutations of *GATA2* are also identified in AML patients, with an incidence varied from 3.6% in patients with French-American-British (FAB) M5 subtype^4^ to 8.1–14.4% in non-selected AML patients^9–11^.

Somatic *GATA2* mutations mainly cluster in the two zinc finger (ZF) domains, which can occupy GATA DNA motif in thousands of genes^9^. The patterns of somatic *GATA2* mutations differ among myeloid diseases. ZF1 mutations predominate in AML, and ZF2 mutations are frequently identified in CML blastic phase^3^. *GATA2* mutations are strongly associated with *CEBPA* double mutations (*CEBPA*^double-mut^)^9,10,12^. However, discrepancies exist among different reports regarding prognostic impact of *GATA2* mutations in AML patients^10,13^. We hypothesize that mutations in different domains of *GATA2* may have distinct impact on clinico-biological features and outcomes in AML patients, like *IDH2* mutations in which *IDH2* R172 is associated with gene mutations and clinical outcomes different from other *IDH* mutations^14^. However, little is known about this issue till now.

In this study, we investigated the clinical and prognostic relevance of mutations in different *GATA2* domains in a large cohort of 693 unselected *de novo* non-M3 AML patients. To our knowledge, this is the first study to show *GATA2* ZF1 mutations are associated with distinct clinical features, gene mutations, and outcomes different from ZF2 mutations. Longitudinal follow-ups were also performed in 419 samples from 124 patients to evaluate the dynamic changes of the mutations. Furthermore, we analyzed the global gene expression profiles in 328 patients to interrogate the possible molecular pathways associated with mutations in different *GATA2* domains.

## Methods and materials

### Subjects

We consecutively enrolled 693 newly diagnosed *de novo* non-M3 AML patients at the National Taiwan University Hospital (NTUH) from 1994 to 2011. Diagnosis and classification of AML were made according to the FAB Cooperative Group Criteria and the 2016 WHO classification^15^. To focus on a more homogeneous group of patients with *de novo* AML, those with antecedent hematological diseases, history of cytopenia, and family history of myeloid neoplasms or therapy-related AML were excluded^16^. Survival analyses were performed in 469 (67.7%) patients who received standard chemotherapy. This study was approved by the Institutional Review Board of the NTUH, and written informed consents were obtained from all participants in accordance with the Declaration of Helsinki.

### Cytogenetics

Chromosomal analyses were performed as described previously^17^. Karyotypes were classified using Medical Research Council (MRC) risk groups^18^.

### Mutation analysis

Mutation analysis of *GATA2* exons 2–6^12^ and 20 other genes, including *FLT3*-ITD^19^, *FLT3*-TKD^19^, *NRAS*^19^, *KRAS*^19^, *KIT*^19^, *PTPN11*^20^*, CEBPA*^21^, *RUNX1*^22^, *MLL*-PTD^23^, *ASXL1*^24^, *IDH1*^25^, *IDH2*^25^, *TET2*^26^, *DNMT3A*^16^, *SF3B1*^27^, *SRSF2*^27^, *U2AF1*^27^, *NPM1*^28^, *WT1*^29^, *TP53*^30^, and *ETV6*^31^ were performed by Sanger sequencing as previously described for patients (*n* = 455) diagnosed from 1994 to 2007. For patients (*n* = 238) diagnosed after 2008, Ion torrent next-generation sequencing (NGS) (Thermo Fisher Scientific, MA, USA) was performed^32^. Serial analyses of mutations at diagnosis, complete remission (CR), and relapse were performed in 419 samples from 124 patients by targeted NGS using TruSight Myeloid Panel (Illumina, San Diego, CA, USA). HiSeq platform (Illumina) was used for sequencing with a median reading depth of 12,000× ^32^.

### Functional annotation analysis of *GATA2* mutation-regulated genes

We analyzed the differentially expression genes associated with *GATA2* mutations by the knowledge-based Ingenuity Pathway Analysis (IPA) (Qiagen, Redwood City, CA) software for associated functions. We also used Gene Set Enrichment Analysis (GSEA) software to investigate systematic enrichments of *GATA2* mutation-governed expressional profile in biological functions^33^. Statistical significance of the degree of enrichment was assessed by a 1000-time random permutation test.

### Statistical analysis

The discrete variables were compared using the *χ*^2^ tests, but if the expected values of contingency tables were <5, Fisher’s exact test was used. Mann–Whitney *U* tests were used to compare continuous variables and medians of distributions. Overall survival (OS) was measured from the date of first diagnosis to the date of last follow-up or death from any cause. Disease-free survival (DFS) was measured from the date of diagnosis until treatment failure, relapse from CR, or death from any cause, whichever occurred first. To ameliorate the influence of hematopoietic stem cell transplantation (HSCT) on survival, DFS and OS were censored at the time of HSCT in patients receiving the treatment^34^. Multivariate Cox proportional hazard regression analysis was used to investigate independent prognostic factors for OS and DFS. A *P* value <0.05 was considered statistically significant. All statistical analyses were performed with the SPSS 18 (SPSS Inc., Chicago, IL, USA) and StatsDirect (Cheshire, England, UK).

## Results

### *GATA2* mutations in patients with AML

**Fig. 1:**
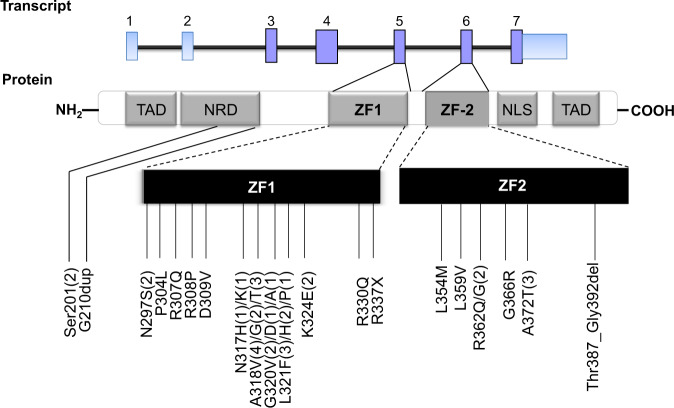
Patterns and locations of the 44 *GATA2* mutations

Excluding two single-nucleotide polymorphisms (A164T, M400T)^35^ and eight missense mutations (N114T, M223I, P250A, A256V, L315P, C319F, V369A, S429T) with unknown biologic significance (because they were not reported previously and could not be verified because of lack of matched bone marrow samples in CR), we identified 44 distinct *GATA2* mutations in 43 (6.2%) of 693 patients (Fig. 1). Forty *GATA2* mutations were missense mutations. The other four were in-frame deletion or duplication: p.Ser201*(c.598_599insG) in two, p.Thr387_Gly392del (c.1160_1177delCCATGAAGAAGGAAGGGA) and G210dup (c.631_632insGCG) in one each. With regard to the functional sites, 31 mutations were clustered in the highly conserved N-terminal ZF domain (ZF1 domain), and other 10 mutations were within C-terminal ZF domain (ZF2 domain). The remaining three mutations scattered outside of the ZF domains. The most common mutations were A318V (*n* = 4), followed by L321F and A318T (*n* = 3 each). p.Ser201*(c.598_599insG), N297S, A318G, G320V, L321H, and K324E occurred in two patients each. All other mutations were detected in only one patient each (Table 1). Only one patient had two *GATA2* mutations (patient no. 20). All mutations were heterozygous. The mutant burden ranged from 4.89 to 52% with a median of 39.07% in ZF1 mutations, and from 10.74 to 50.26% with a median of 36.16% in ZF2 mutations.

**Table 1:** The mutation patterns in 43 patients with *GATA2* mutations at diagnosis

				*GATA2* mutations			
UPN	Age/sex	Karyotype	Location	DNA change	Mutant burden (%)	Protein change	Other mutations
1	29F	CN	ZF1	c.953C>T	52	A318V	*CEBPA*^dm^, *FLT3*-ITD, *NRAS*
2	40M	CN	ZF1	c.961C>T	49.37	L321F	*CEBPA*^dm^, *NRAS*
3	65F	t(3;3)(q21;q26),del(12)(p11p13)	ZF1	c.890A>G	49.04	N297S	*NRAS*, *ASXL1*
4	36M	CN	ZF1	c.961C>T	47.42	L321F	*CEBPA* ^dm^
5	37M	−Y	ZF1	c.959G>T	47.19	G320V	*CEBPA* ^dm^
6	36M	CN	ZF1	c.970A>G	46.14	K324E	*CEBPA*^dm^, *NRAS*
7	27M	CN	ZF1	c.953C>G	45.45	A318G	*CEBPA* ^dm^
8	78F	+8	ZF1	c.1009C>T	45.3	R337X	*FLT3*-ITD, *NRAS*, *IDH2*, *SRSF2*
9	42M	t(3;3)(q21;q26)/46,idem,add(17)(p13)	ZF1	c.959G>A	44.62	G320D	*ASXL1*, *U2AF1*
10	34M	CN	ZF1	c.962T>A	43.97	L321H	*CEBPA*^sm^, *NRAS*, *KIT*, *IDH2*, *DNMT3A*
11	20F	CN	ZF1	c.989G>A	43.14	R330Q	*CEBPA*^dm^, *ASXL1*
12	32M	CN	ZF1	c.952G>A	42.99	A318T	*CEBPA*^dm^, *KIT*
13	39M	CN	ZF1	c.911C>T	42.41	P304L	*MLL*, *TET2*
14	43M	CN	ZF1	c.923G>C	41.21	R308P	*CEBPA*^dm^, *NRAS*
15	18M	del(9)(q22q34)	ZF1	c.926A>G	39.07	D309V	*CEBPA* ^sm^
16	36F	CN	ZF1	c.920G>A	39.06	R307Q	*CEBPA*^dm^, *NRAS*
17	31F	CN	ZF1	c.970A>G	37.98	K324E	*CEBPA* ^dm^
18	55M	CN	ZF1	c.952G>A	32.72	A318T	*CEBPA* ^dm^
19	69F	CN	ZF1	c.953C>T	30.26	A318V	*CEBPA* ^dm^
20	57M	CN	ZF1	c.962T>A c.949A>C	23.94	L321H, N317H	*CEBPA*^dm^, *TET2*
21	51M	+21	ZF1	c.953C>T	23.48	A318V	*CEBPA*^dm^, *RUNX1*
22	39F	46,XX,der(3)t(3;17)(q26;q21),t(16;17)(p11;q11)	ZF1	c.962T>C	20.48	L321P	*SF3B1*
23	82M	CN	ZF1	c.951T>A	20.46	N317K	*RUNX1*, *SF3B1*
24	19F	CN	ZF1	c.959G>C	18.41	G320A	*CEBPA*^dm^, *FLT3-*TKD
25	59M	CN	ZF1	c.953C>T	18.15	A318V	*CEBPA*^dm^, *NRAS*
26	29M	CN	ZF1	c.961C>T	17.58	L321F	*CEBPA* ^sm^
27	50M	CN	ZF1	c.959G>T	13.81	G320V	*CEBPA*^dm^, *U2AF1*
28	54M	CN	ZF1	c.953C>G	10.81	A318G	*CEBPA* ^dm^
29	22F	del(9q)	ZF1	c.952G>A	6.02	A318T	*CEBPA* ^dm^
30	78M	CN	ZF1	c.890A>G	4.89	N297S	*PTPN11*, *DNMT3A*
31	76M	CN	ZF2	c.1075T>G	50.26	L359V	*RUNX1*
32	53F	CN	ZF2	c.1085G>A	48.68	R362Q	*ASXL1*, *IDH2*, *DNMT3A*
33	28F	CN	ZF2	c.1114G>A	46.82	A372T	*NPM1*
34	69F	CN	ZF2	c.1096G>A	46.33	G366R	*NPM1*
35	18F	CN	ZF2	c.1114G>A	39.8	A372T	*NPM1*, *PTPN11*
36	20M	CN	ZF2	c.1084C>G	23.05	R362G	*CEBPA*^sm^, *ASXL1*
37	40F	t(7;11)	ZF2	c.1114G>A	21.36	A372T	*FLT3-*ITD, *NRAS*
38	60M	−Y	ZF2	c.1084C>G	32.52	R362G	*-*
39	32F	+10	ZF2	c.1160_1177delCCATGAAGAAGGAAGGGA	17.59	Thr387_Gly392del	*CEBPA*^dm^, *NRAS*
40	80F	CN	ZF2	c.1061C>T	10.74	T354M	*CEBPA*^dm^, *FLT3*-ITD
41	71M	del(12)(p12p13), −7		c.598_599insG	35.31	Ser201	*PTPN11*, *RUNX1*, *ASXL1*
42	68F	CN		c.598_599insG	34.1	Ser201	*FLT3-*ITD, *RUNX1*, *MLL*
43	76M	CN		c.631_632insGCG	40.36	G210dup	*TP53*

*UPN* unique patient number, *CEBPA*^dm^ *CEBPA* double mutation, *CN* cytogenetically normal, *ZF* zinc finger

### Correlation of *GATA2* mutations with clinical and laboratory features

Table 2 depicted the clinical characteristics of patients with and without *GATA2* mutations. ZF1-mutated patients were younger (median, 39 years vs. 55 years, *P* = 0.004), and had higher incidence of FAB M1 subtype (56.7% vs. 22.1%, *P* < 0.0001), but lower incidence of FAB M4 subtype (3.3% vs. 28.1%, *P* = 0.003) than *GATA2-*wild patients. ZF1-mutated patients also had a higher incidence of FAB M1 subtype than ZF2-mutated patients (*P* = 0.044). The patients with ZF2 mutations showed similar clinical features to the *GATA2-*wild group, including peripheral white blood cell counts (median, 47.3 vs. 18.7 k/µL), incidences of FAB M1 subtype (20% vs. 22.1%), and M4 subtype (20% vs. 28.1%).

**Table 2:** Comparison of clinical and laboratory features between AML patients with *GATA2* ZF1 domain and ZF2 domain mutations

Variables	*GATA2-*wild (*n* = 650)	*GATA2* mutations (*n* = 43)	*P* value^a^	ZF1 domain mutations (*n* = 30)	*P* value^b^	ZF2 domain mutations (*n* = 10)	*P* value^c^
Sex^d^			0.876		0.291		0.112
Male	370 (56.9)	25 (58.1)		20 (66.7)		3 (30)	
Female	280 (43.1)	18 (41.9)		10 (33.3)		7 (70)	
Age (year)^e^	55 (15–94)	40 (18–82)	0.017	39 (18–82)	0.004	47 (18–80)	0.365
Lab data^e^							
WBC (k/μL)	18.7 (0.12–423)	21.2 (1.23–627.8)	0.200	23.4 (1.33–627.8)	0.195	47.3 (1.23–212.7)	0.494
Hb (g/dL)	8.1 (2.9–16.2)	8.1 (4.2–13.2)	0.704	8.1 (4.4–12.5)	0.436	7.4 (4.2–13.2)	0.311
Platelet (k/μL)	47 (3–802)	45 (6–1017)	0.565	47 (6–1017)	0.937	47 (11–119)	0.606
PB Blast(k/μL)	7.33 (0–371.9)	9.09 (0–456.7)	0.077	11.3 (0.06–456.7)	0.067	29.9 (0–140.7)	0.358
LDH (U/L)	859 (206–15000)	917 (299–4220)	0.575	970 (327–4220)	0.385	1029 (394–2970)	0.629
FAB^d^							
M0	16 (2.5)	2 (4.7)	0.309	2 (6.7)	0.186	0 (0)	>0.999
M1	144 (22.1)	21 (48.8)	<0.0001	17 (56.7)	<0.0001	2 (20)	>0.999
M2	239 (36.8)	17 (39.5)	0.716	10 (33.3)	0.703	6 (60)	0.186
M4	183 (28.1)	3 (7.0)	0.002	1 (3.3)	0.003	2 (20)	0.734
M5	31 (4.8)	0 (0)	0.248	0 (0)	0.633	0 (0)	>0.999
M6	27 (4.2)	0 (0)	0.403	0 (0)	0.625	0 (0)	>0.999
Unclassified	10 (1.5)	0 (0)	>0.999	0 (0)	>0.999	0 (0)	>0.999
2016 WHO classification^d^							
t(8;21)	57 (8.7)	0 (0)	0.041	0 (0)	0.165	0 (0)	>0.999
Inv(16)	27 (4.2)	0 (0)	0.403	0 (0)	0.625	0 (0)	>0.999
t(9;11)	9 (1.4)	0 (0)	>0.999	0 (0)	>0.999	0 (0)	>0.999
t(6;9)	3 (0.5)	0 (0)	>0.999	0 (0)	>0.999	0 (0)	>0.999
Inv(3)	1 (0.2)	2 (4.6)	0.011	2 (6.7)	0.005	0 (0)	>0.999
t(1;22)	0 (0)	0 (0)	–	0 (0)	–	0 (0)	–
* CEBPA* ^dm^	43 (6.6)	22 (51.2)	<0.0001	20 (66.7)	<0.0001	2 (20)	0.144
* NPM1*	139 (21.3)	3 (7.0)	0.023	0 (0)	0.005	3 (30)	0.455
* RUNX1*	73 (11.2)	4 (9.3)	>0.999	1 (3.3)	0.237	1 (10)	>0.999
BCR-ABL	1 (0.2)	0 (0)	>0.0999	0 (0)	>0.999	0 (0)	>0.999
MRC	93 (14.3)	0 (0)	0.008	0 (0)	0.025	0 (0)	0.372
AML, NOS	204 (31.4)	12 (27.9)	0.633	7 (23.3)	0.351	4 (40)	0.516
Induction response^f^	431	38		27		9	
Complete remission	323 (74.9)	29 (76.3)	0.851	23 (85.2)	0.230	5 (60)	0.241
Induction death	32 (7.4)	1 (2.6)	0.503	0 (0)	0.243	1 (10)	0.508
Relapse	161 (49.8)	9 (31)	0.052	8 (34.8)	0.163	1 (16.7)	0.371

*CEBPA*^sm^ *CEBPA* single mutation, *CEBPA*^dm^ *CEBPA* double mutation, *MRC* myelodysplasia-related change, *NOS* not otherwise specified, *PB* peripheral blood

^a^*GATA2*-mutated patients vs. *GATA2* wild-type patients

^b^*GATA2* ZF1-mutated patients vs. *GATA2* wild-type patients

^c^*GATA2* ZF2-mutated patients vs. *GATA2* wild-type patients

^d^Number of patients (%)

^e^Median (range)

^f^Only the 469 patients, including 27 with *GATA2 ZF1* domain mutations, nine with *GATA2 ZF2* domain mutations, and 431 without, who received conventional intensive induction chemotherapy and then consolidation chemotherapy if CR was achieved, as mentioned in the text, were included in the analysis

### Association of *GATA2* mutations with cytogenetics abnormalities

Chromosome data were available in 669 patients at diagnosis, including 43 *GATA2*-mutated and 626 *GATA2*-wild patients (Supplementary Table 1). Totally, *GATA2* mutations were closely associated with intermediate-risk cytogenetics. Compared to *GATA2*-wild patients, ZF1-mutated patients had more intermediate-risk cytogenetics (100% vs. 70.9%, *P* < 0.0001), normal karyotype (73.3% vs. 46.5%, *P* = 0.004), and t(3;3) (6.7% vs. 1.0%, *P* = 0.048), but less favorable-risk (0% vs. 13.6%, *P* = 0.024) or unfavorable-risk cytogenetics (0% vs. 15.5%, *P* = 0.014). There was no association of ZF1 mutations with other chromosomal abnormalities, including +8, +11, +13, and +21.

### Association of *GATA2* mutations with other molecular alterations

To investigate the interaction of *GATA2* ZF1 and ZF2 mutations with other genetic alterations in the pathogenesis of adult AML, a complete mutational screening of 20 other genes was performed. Only ZF1-mutated patients had a significantly higher frequency of *CEBPA*^double-mut^ (66.7% vs. 6.7%, *P* < 0.0001) than wild-type patients, but not ZF2-mutated patients (Table 3). ZF1-mutated patients had lower frequencies of *NPM1* mutations (0% vs. 22%, *P* = 0.004) and *FLT3*-ITD (4% vs. 19.9%, *P* = 0.024) than wild-type patients. In contrast, ZF2-mutated patients had similar frequencies of *NPM1* mutations (30%) and *FLT3*-ITD (20%) to those with wild type of *GATA2*. Both ZF1 and ZF2 mutations were mutually exclusive with *KRAS*, *WT1*, *IDH1*, *TP53*, and *ETV6* mutations (Table 3).

**Table 3:** Comparison of other genetic alterations between AML patients according to *GATA2* mutation domain

Mutation	Total pts examined	Pts with the other gene mutations (%)					*P* value^a^	*P* value^b^	*P* value^c^
		Whole cohort	*GATA2* wt pts	*GATA2* mutated pts	ZF1	ZF2			
*FLT3*-ITD	685	19.3	19.9	9.3	4.0	20	0.087	0.024	>0.999
*FLT3-*TKD	690	8.8	9.2	2.4	3.3	0	0.248	0.508	>0.999
*NRAS*	691	15.5	14.8	26.8	30	25	0.038	0.035	0.340
*KRAS*	688	3.6	3.9	0	0	0	0.391	0.620	>0.999
*PTPN11*	658	5	4.8	7.7	3.6	12.5	0.436	>0.999	0.335
*KIT*	690	4.8	4.8	4.9	6.7	0	>0.999	0.652	>0.999
*WT1*	688	6.8	7.3	0	0	0	0.103	0.257	>0.999
*NPM1*	693	21.1	22	7	0	30	0.019	0.004	0.467
*CEBPA*	689	14.2	11.1	60.5	76.7	30	<0.0001	<0.0001	0.095
*CEBPA*^*dm*^	689	9.4	6.7	51.2	66.7	20	<0.0001	<0.0001	0.146
*RUNX1*	684	14	14.2	11.9	6.7	11.1	0.682	0.413	>0.999
*MLL/*PTD	636	5.7	5.7	5.1	3.6	0	>0.999	>0.999	>0.999
*ASXL1*	691	14	14	14	10	20	0.987	0.786	0.640
*IDH1*	690	6.4	6.8	0	0	0	0.101	0.250	>0.999
*IDH2*	691	12.7	13.1	7.1	6.7	11.1	0.262	0.410	>0.999
*TET2*	670	11.9	12.4	4.9	6.9	0	0.212	0.562	0.610
*DNMT3A*	685	17.4	18	7.3	6.9	11.1	0.080	0.124	>0.999
*TP53*	685	7.7	8.1	2.4	0	0	0.241	0.158	>0.999
*ETV6*	649	0.9	0.9	0	0	0	>0.999	>0.999	>0.999
SF	653	11.8	11.7	12.5	17.9	0	0.802	0.366	0.608

Pts patients, *CEBPA*^*dm*^ *CEBPA*^*double-mutation*^, *SF* splicing factors, including *SF3B1*, *SRSF2*, and *U2AF1*

^a^*GATA2*-mutated patients vs. *GATA2* wild-type patients

^b^*GATA2* ZF1-mutated patients vs. *GATA2* wild-type patients

^c^*GATA2* ZF2-mutated patients vs. *GATA2* wild-type patients

### Impact of different *GATA2* domains mutations on treatment response and clinical outcomes

Of the 469 AML patients, including 27 *GATA2* ZF1-mutated and nine *GATA2* ZF2-mutated patients, undergoing conventional intensive induction chemotherapy, 352 (75.1%) patients achieved a CR. The CR rate was 85.2% in ZF1-mutated patients and 60% in ZF2-mutated patients (Table 2). The relapse rate was similar between the two groups.

**Fig. 2:**
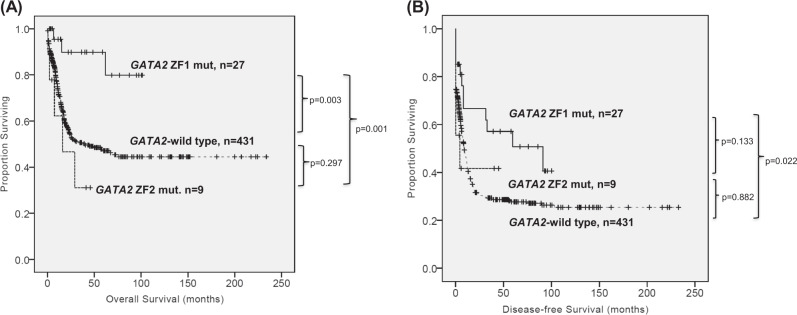
**Kaplan–Meier survival curves for OS (a) and DFS (b) stratified by the** ***GATA2*** **mutation status and the sites of mutations in 467 AML patients who received standard intensive chemotherapy.** Patients with *GATA2* ZF1 mutations had a significantly better OS (5-year survival rate, 72% vs. 43%, *P* = 0.003) and DFS than *GATA2-*wild patients (median, 91.2 vs. 8.8 months, *P* = 0.022). Patients with *GATA2* ZF2 mutations had similar OS (5-year survival rate, 31%, *P* = 0.297) and DFS (median, 4.4 months, *P* = 0.882) as the wild-type group. ZF1 mutations were also associated with better OS compared with ZF2 mutations (*P* = 0.001)

**Fig. 3:**
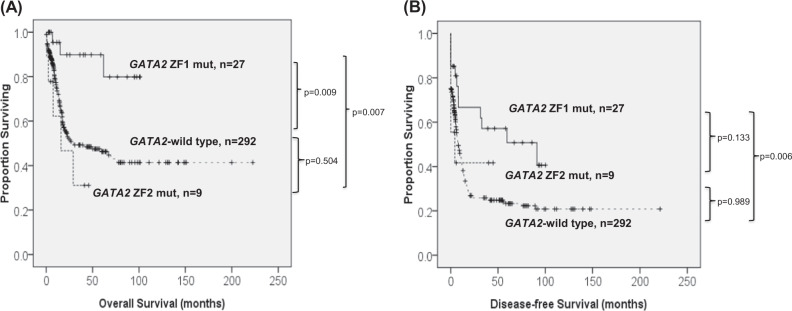
**Kaplan–Meier survival curves for OS (a) and DFS (b) stratified by the** ***GATA2*** **mutation status and the sites of mutations in 328 intermediate-risk cytogenetics patients who received standard intensive chemotherapy.** Patients with *GATA2* ZF1 mutations had a significantly better OS (5-year survival rate, 72% vs. 39%, *P* = 0.009) and DFS (median, 91.2 vs. 7.8 months, *P* = 0.006) than *GATA2-*wild patients. Patients with *GATA2* ZF2 mutations had similar OS and DFS as the wild-type group (*P* = 0.504, *P* = 0.989, respectively). ZF1 mutations were also associated with a longer OS (5-year survival rate, 72% vs. 31%, *P* = 0.007) and a trend toward longer DFS (median, 91.2 vs. 4.4 months, *P* = 0.133) compared with ZF2 mutations

With a median follow-up time of 78.6 months (ranges, 0.1–236 months), patients with *GATA2* mutations as a whole had a trend of longer OS (5-year survival rate, 56% vs. 43%, *P* = 0.078) and DFS (median, 32.9 vs. 8.8 months, *P* = 0.091) than those without *GATA2* mutations (Supplementary Figure 1). Focusing on the prognostic implication of mutation sites, patients with *GATA2* ZF1 mutations had a significantly better OS (5-year survival rate, 72% vs. 43%, *P* = 0.003) and DFS than *GATA2-*wild patients (median, 91.2 vs. 8.8 months, *P* = 0.022) (Fig. 2). In contrast, patients with *GATA2* ZF2 mutations had similar OS (5-year survival rate, 31%, *P* = 0.297) and DFS (median, 4.4 months, *P* = 0.882) as the *GATA2-*wild group. Intriguingly, ZF1 mutations were also associated with better OS compared with ZF2 mutations (*P* = 0.001) (Fig. 2). In intermediate-risk cytogenetics group, ZF1-mutated patients had significantly superior OS (5-year survival rate, 72% vs. 39%, *P* = 0.009) and DFS (median, 91.2 vs. 7.8 months, *P* = 0.006) than *GATA2-*wild patients, and a longer OS (5-year survival rate, 72% vs. 31%, *P* = 0.007) and a trend toward longer DFS (median, 91.2 vs. 4.4 months, *P* = 0.133) than ZF2-mutated patients (Fig. 3). The finding also held true in normal karyotype subgroup (Supplementary Figure 2). Multivariate analysis demonstrated that ZF1 mutation was an independent favorable prognostic factor for OS (HR 0.207, 95% CI 0.066–0.652, *P* = 0.007) and DFS (HR 0.529, 95% CI 0.295–0.948, *P* = 0.032) irrespective of age, white blood cell counts, cytogenetics, *NPM1*, and *FLT3-*ITD status. However, the prognostic independence of ZF1 mutation was lost if we included *CEBPA*^double-mut^ as a covariable (Supplementary Table 2). We could not find the survival difference stratified by the degree of mutational burden in either ZF1 or ZF2-mutated patients (data not shown). Allo-HSCT in CR1 for ZF1-mutated patients did not offer survival benefit compared to postremission chemotherapy alone (data not shown).

**Fig. 4:**
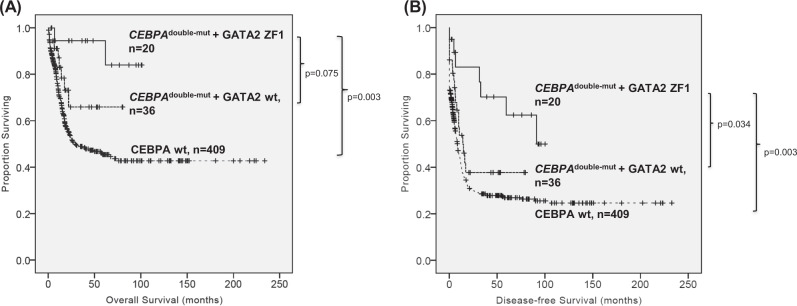
**Comparison of OS (a) and DFS (b) among** ***CEBPA***^double-mut^**/*GATA2*** **ZF1-mutated,** ***CEBPA***^double-mut^**/*GATA2-*wild and** ***CEBPA-*wild AML patients who received standard intensive chemotherapy**. C*EBPA*^double-mut^ patients with *GATA2* ZF1 mutations had a trend of longer OS (5-year survival rate, 76% vs. 68%, *P* = 0.075) and a significantly longer DFS (median, 91.2 vs. 14.0 months, *P* = 0.034) that those with wild-type *GATA2*. The small number of ZF2-mutated patients in C*EBPA*^double-mut^ patients did not allow statistically meaningful correlations

In *CEBPA*^double-mut^ subgroup, *GATA2* ZF1*-*mutated patients had a trend of longer OS (5-year survival rate, 76% vs. 68%, *P* = 0.075) and a significantly longer DFS (median, 91.2 vs. 14.0 months, *P* = 0.034) than *GATA2*-wild patients (Fig. 4). ZF1 mutations allowed further refinement of the clinical outcome of *CEBPA*^double-mut^ patients. The small number of ZF2-mutated patients (*n* = 3) in this group did not allow statistically meaningful correlations.

### Sequential studies of *GATA2* mutations in AML patients

*GATA2* mutations were serially studied in 419 samples from 124 patients who had ever obtained a CR and had available samples for study, including 19 patients with and 105 patients without *GATA2* mutations at diagnosis (Table 4). Among the 19 *GATA2*-mutated patients who had paired samples, all lost the original *GATA2* mutations at remission. Five of the six patients regained the original *GATA2* mutations at first relapse, but one (no. 27) lost the mutation. In the former five patients, the mutation burden, compared to that at diagnosis, was increased in one patient (no. 25), decreased in two (nos. 13 and 16), and stable in the remaining two (nos. 5 and 9). One patient (no. 9) retained the co-occurring *ASXL1* mutations at CR status. Among the 105 patients who had no *GATA2* mutations at diagnosis, four patients (nos. 44, 45, 46, and 47) acquired novel *GATA2* mutations at relapse (Table 4).

**Table 4:** Sequential studies in the AML patients with *GATA2* mutations^a^

UPN	Interval^b^ (months)	Status	*GATA2* mutations	Allele burden	Other mutations
1		Initial	Ala318Val	52	*CEBPA*, *FLT3-ITD*, *NRAS*
	0.9	CR1	−	0	−
4		Initial	Leu321Phe	47.42	*CEBPA*
	1.3	CR1	−	0	*−*
5		Initial	Gly320Val	47.19	*CEBPA*
	6.6	CR1	−	0	−
	27.1	Relapse1	Gly320Val	43.1	*CEBPA*
	1.0	CR2	−	0	−
6		Initial	Lys324Glu	46.14	*CEBPA*, *NRAS*
	0.9	CR1	−	0	*−*
7		Initial	Ala318Gly	45.45	*CEBPA*
	0.9	CR1	−	0	*−*
9		Initial	Gly320Asp	44.62	*ASXL1*, *U2AF1*
	3.2	CR1	−	0	*ASXL1*
	6.5	Relapse1	Gly320Asp	43.2	*ASXL1*
12		Initial	Ala318Thr	42.99	*CEBPA*, *KIT*
	0.9	CR1	−	0	*−*
13		Initial	Pro304Leu	42.41	*MLL*, *TET2*
	3.5	CR1	−	0	*−*
	6.3	Relapse1	Pro304Leu	3.2	*−*
14		Initial	Arg308Pro	41.21	*CEBPA*, *NRAS*
	1.4	CR1	−	0	*-*
16		Initial	Arg307Gln	39.06	*CEBPA*, *NRAS*
	3.0	CR1	−	0	*−*
	34.7	Relapse1	Arg307Gln	27	*CEBPA*
18		Initial	Ala318Thr	32.72	*CEBPA*
	2.1	CR1	−	0	*−*
20		Initial	Leu321His, Asn317His	11.3	*CEBPA*, *TET2*
				23.94	
	1.4	CR1	−	0, 0	*−*
21		Initial	Ala318Val	23.48	*CEBPA*, *RUNX1*
	1.0	CR1	−	0	*−*
24		Initial	Gly320Ala	18.41	*CEBPA*, *FLT3*-TKD
	0.9	CR1	−	0	*−*
25		Initial	Ala318Val	18.15	*NRAS*, *CEBPA*
	1.2	CR1	−	0	−
	12.0	Relapse1	Ala318Val	43.5	*CEBPA*
27		Initial	Gly320Val	13.81	*CEBPA*, *U2AF1*
	1.0	CR1	−	0	−
	3.5	Relapse1	−	0	*CEBPA*
	11.7	CR2	−	0	−
	5.9	Relapse2	−	0	*CEBPA*
29		Initial	Ala318Thr	6.02	*CEBPA*
	1.0	CR1	−	0	
39		Initial	Thr387_Gly392del	17.59	*CEBPA*, *NRAS*
	1.0	CR1	−	0	*−*
41		Initial	Ser201	35.31	*PTPN11*, *RUNX1*, *ASXL1*
	0.8	CR1	−	0	*−*
44		Initial	−	0	*CEBPA*, *DNMT3A*
	4.5	CR1	−	0	*DNMT3A*
	2.9	Relapse1	Glu180LysfsTer38	7.1	*DNMT3A*
	1.1	CR2	−	0	−
	6.0	Relapse2	−	0	*DNMT3A*
	2.0	CR3	−	0	−
45		Initial	−	0	*DNMT3A*, *NPM1*, *NRAS*, *PTPN11*
	7.3	CR1	−	0	*DNMT3A*
	12.5	Relapse1	Arg307Leu	5.6	*DNMT3A*, *NPM1*
	1.2	CR2	−	0	*DNMT3A*
	13.6	Relapse2	−	0	*DNMT3A*, *NPM1*
46		Initial	−	0	*CEBPA*
	2.9	CR1	−	0	−
	14.2	Relapse1	Leu321Pro	26	*CEBPA*
47	29.0	Initial	−	0	*−*
	1.0	CR1	−	0	−
	15.6	Relapse1	Gly320Asp	15.9	−
			Leu321His	15.1	
	3.6	CR2	−	0	−
	11.8	Relapse2	Leu321His	39.4	*−*
	4.8	CR3	−	0	

*UPN* unique patient number, *CR* complete remission, *ND* not done, “−” negative

^a^The results of serial studies in 101 patients without GATA2 mutation at both diagnosis and relapse were not shown in this table

^b^Interval between the two successive statuses

### *GATA2* expression and biological functions associated with *GATA2* mutations

We analyzed the microarray dataset of 328 patients studied to assess the impact of *GATA2* mutations on gene expression and biological functions. By comparing the mRNA expression profiles between patients with and without *GATA2* mutations, we found *GATA2* expression levels were higher in those with *GATA2* mutations (*P* = 0.003). More specifically, both ZF1 and ZF2 mutations correlated with higher *GATA2* expression level compared to *GATA2* wild-type. *GATA2* mutations were associated with significant differential expression of 159 probes (*t*-test, *P* < 0.05 and >2-fold change). IPA analysis revealed different molecular networks between the *GATA2* ZF1 and ZF2-mutated group (Supplementary Figure 3). We also performed the GSEA analysis to identify biological functions associated with genes significantly enriched in *GATA2*-mutated AML, compared with *GATA2*-wild AML. Three-hundred and thirteen patients with wild-type *GATA2*, 12 patients with *GATA2* ZF1 mutations, and three patients with *GATA2* ZF2 mutations were analyzed. We identified significant underrepresentation of genes hyper-methylated in AML (*P* = 0.006; normalized enrichment score (NES) = −1.49; Supplementary Figure 4A) and genes related to apoptosis (*P* = 0.042; NES = −1.33) in the ZF1-mutated patients compared to *GATA2* wild-type patients. ZF2-mutations were associated with the Gene Oncology term of myeloid leukocyte differentiation (*P* = 0.03; NES = −1.46) (Supplementary Figure 4B). Comparing with ZF2-mutated AML, we identified significant overrepresentation of genes related to myeloid leukocyte differentiation (*P* = 0.042; NES = 1.36) and underrepresentation of genes hyper-methylated in AML (*P* = 0.029; NES = −1.37) in the ZF1-mutated AML.

## Discussion

To the best of our knowledge, this is the first study to explore differences in clinical and biological implications between the *GATA2* ZF1 and ZF2 mutations in AML patients. We found that mutations in different domains were associated with distinct clinical features, co-occurring mutations and outcomes (Supplementary Table 3).

The *GATA2* mutation landscape in adult *de novo* AML differs from that in blastic crisis of CML^3^, familial MDS/AML^4^, and pediatric AML^5^. In adult AML, ZF1 mutations predominate, while ZF2 mutations are reported sporadically^10,36,37^. In concordance with the findings, two-thirds of the 44 distinct *GATA2* mutations in our study were located in the ZF1 domain. We also reported two novel missense mutations in ZF2 domain (L359V and G366R) that had not been reported before in adult *de novo* AML patients, but ever identified in blastic crisis of CML.

AML with *CEBPA*^double-mut^ has been included as a definite entity in the 2016 WHO Classification of Myeloid Neoplasms^15^. It is well established that *GATA2* mutations frequently co-occur with *CEBPA*^double-mut^ with an incidence of 18–41%^9,10,12^ and the two proteins show direct protein–protein interaction^38^. Further study revealed *GATA2* ZF1 mutants, but not the ZF2 L359V that is commonly seen at the progression of CML to blast crisis, had reduced capacity to enhance *CEBPA*-dependent activation of transcription^9^. Based on this functional study and the frequent co-occurrence of *CEBPA*^double-mut^ and ZF1 mutations, but not ZF2 mutations, in AML patients, it is possible that *GATA2* ZF1 mutations and *CEBPA*^double-mut^ interact together to induce leukemogenesis. In addition, we found ZF1 mutations were associated with lower incidences of *NPM1* mutations and *FLT3*-ITD than wild-type *GATA2*, different from ZF2 mutations as ZF2-mutated patients had similar incidences of these two mutations to those in *GATA2*-wild patients. *GATA2* ZF1 and ZF2 mutations may induce AML through different oncogenic mechanisms and have distinct impact on clinical outcomes. Truly, in this study, we demonstrated that patients with *GATA2* ZF1 mutations had a significantly longer OS than ZF2-mutated patients in total cohort, as well as in patients with intermediate-risk cytogenetics and normal karyotype.

The prognostic impact of *GATA2* mutations in *CEBPA*^double-mut^ patients was conflicting^12,13,37,39^. Greif et al. and Theis et al. found that G*ATA2* mutations did not impact clinical outcome in *CEBPA*^double-mut^ patients. On the contrary, *GATA2* mutations correlated with improved survival among *CEBPA*^double-mut^ patients in other reports^12,13^. In a study of Theis et al., 31 (74%) of *GATA2* mutations were detected in ZF1 domain, and 11 (26%) in ZF2 domain. They did not show different clinical outcomes with respect to *GATA2* ZF1 and ZF2 mutations in a cohort with both *CEBPA*^double-mut^ and *CEBPA*^single-mut^ patients^39^. We were the first to investigate the prognostic implication of *GATA2* ZF1 mutations in *CEBPA*^double-mut^ patients and showed its association with a better DFS and a trend of longer OS than wild-type *GATA2* among the *CEBPA*^double-mut^ subgroup.

The poor prognostic impact of *GATA2* ZF2 mutations was also witnessed in blast crisis CML patients as in *de novo* AML patients shown in this study^4^. The reason that ZF1 and ZF2 mutations had different survival impacts on de novo AML patients might be partially explained by their difference in association with *CEBPA*^double-mut^, and by different oncogenic mechanisms. Further studies are warranted to explore the underlying mechanisms of the differences.

The study also recruited the largest number of *de novo* AML patients for sequential analyses of *GATA2* mutations by NGS during clinical follow-ups. The original mutations in all 19 *GATA2-*mutated patients were lost at remission status, confirming them to be truly somatic mutations. We showed *GATA2* mutation was not stable during disease evolution. One (no. 27) of the six patients with *GATA2* mutations at diagnosis lost the mutation at relapse. Among the 105 patients who had no *GATA2* mutations at diagnosis, four (nos. 44, 45, 46, 47) acquired novel *GATA2* mutations at relapse. The four mutations were all ZF1 mutations.

In conclusion, *GATA2* ZF1 mutations, but not ZF2 mutations, are closely associated with *CEBPA*^double-mut^, and inversely correlated with *NPM1* mutations and *FLT3*-ITD. The two *GATA2* ZF domain mutations have different impacts on OS in AML patients. *GATA2* ZF1 mutations also affect clinical outcome in *CEBPA*^double-mut^ patients. Incorporation of *GATA2* ZF1, not ZF2 mutations, allows further refinement of the WHO Classification in the specific entity of AML with *CEBPA*^double-mut^.

## Electronic supplementary material

Supplementary data
