# Supplementary material for: GATA2 zinc finger 1 mutations are associated with distinct clinico-biological features and outcomes different from GATA2 zinc finger 2 mutations in adult acute myeloid leukemia
Source: Blood Cancer J. 2018 Aug 31;8(9):87. doi: 10.1038/s41408-018-0123-2 (PMC6127202; doi:10.1038/s41408-018-0123-2)
Supplement: Supplementary file 1 — Supplementary data [file 41408_2018_123_MOESM1_ESM.docx]

**Supplementary Figure 1**

**Kaplan–Meier survival curves for OS (A) and DFS (B) stratified by the status of *GATA2* mutations in total 469 AML patients who received standard intensive chemotherapy**

(A) (B)

***GATA2*-mutated, n=38**

***GATA2*-wild type, n=431**

**P=0.078**

**P=0.091**

***GATA2*-wild type, n=431**

***GATA2*-mutated, n=38**

**Supplementary Figure 2**

**Kaplan–Meier survival curves for OS (A), DFS (B) stratified by the *GATA2* mutation status and the sites of mutations in 233 normal karyotype patients who received standard intensive chemotherapy**

Patients with *GATA2* ZF1 mutations had significantly better OS and DFS than those with wild-type (P=0.003, P=0.009, respectively). Patients with *GATA2* ZF2 mutations had similar OS and DFS as the wild-type group (P=0.504, P=0.746, respectively). ZF1 mutations were also associated with a better OS (P=0.001) and a trend toward better DFS compared with ZF2 mutations (P=0.133).

1. (B)

**
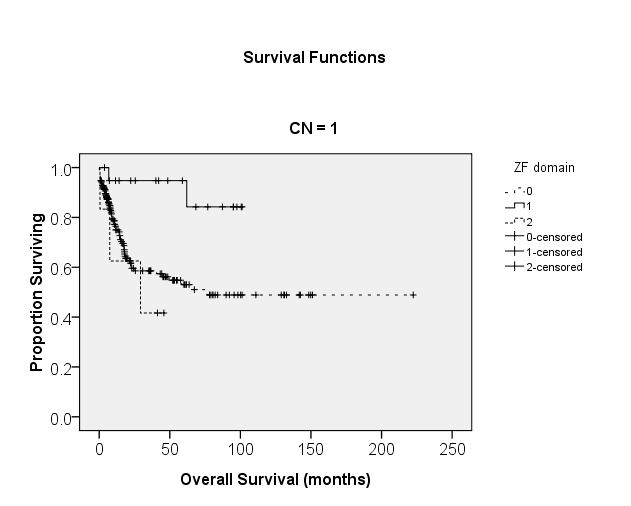
**


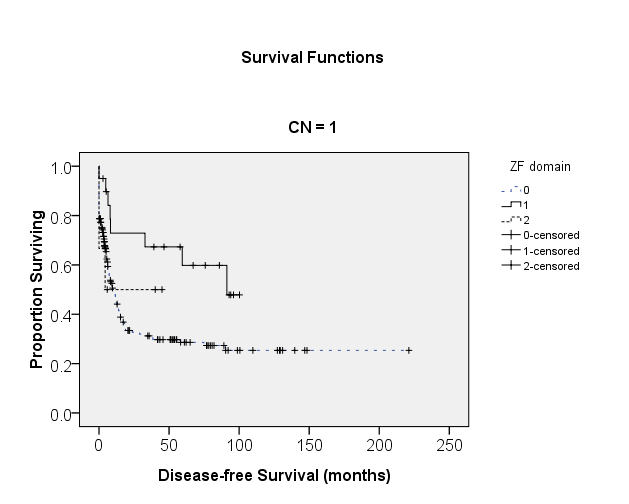


***GATA2*-wild type, n=207**

***GATA2* ZF2 mut, n=6**

***GATA2* ZF1 mut, n=20**

***GATA2*-wild type, n=207**

***GATA2* ZF2 mut, n=6**

***GATA2* ZF1 mut, n=20**

**Supplementary Figure 3**

**Mechanistic networks of (A) *GATA2* ZF1 mutations, (B) *GATA2* ZF2 mutations generated by IPA.**

**(A)**

**
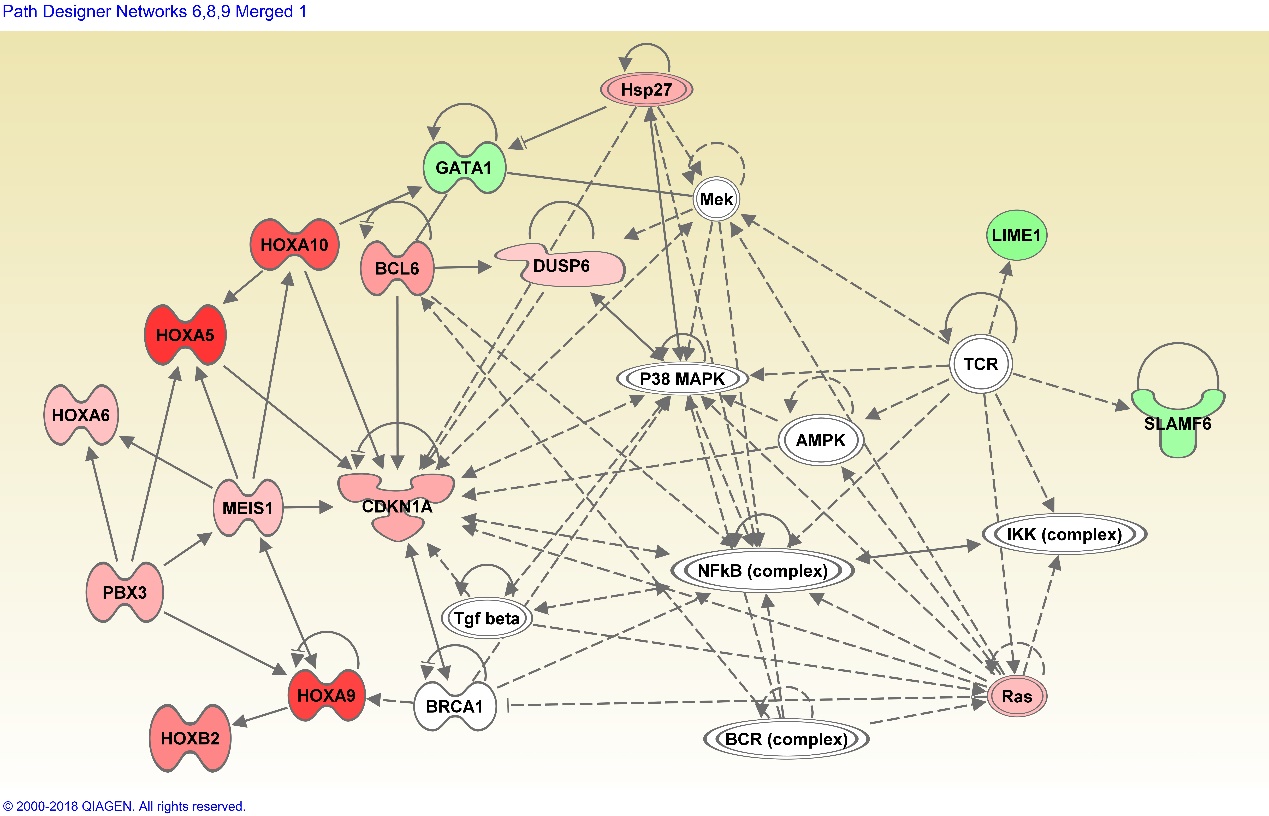
**

**(B)**

**
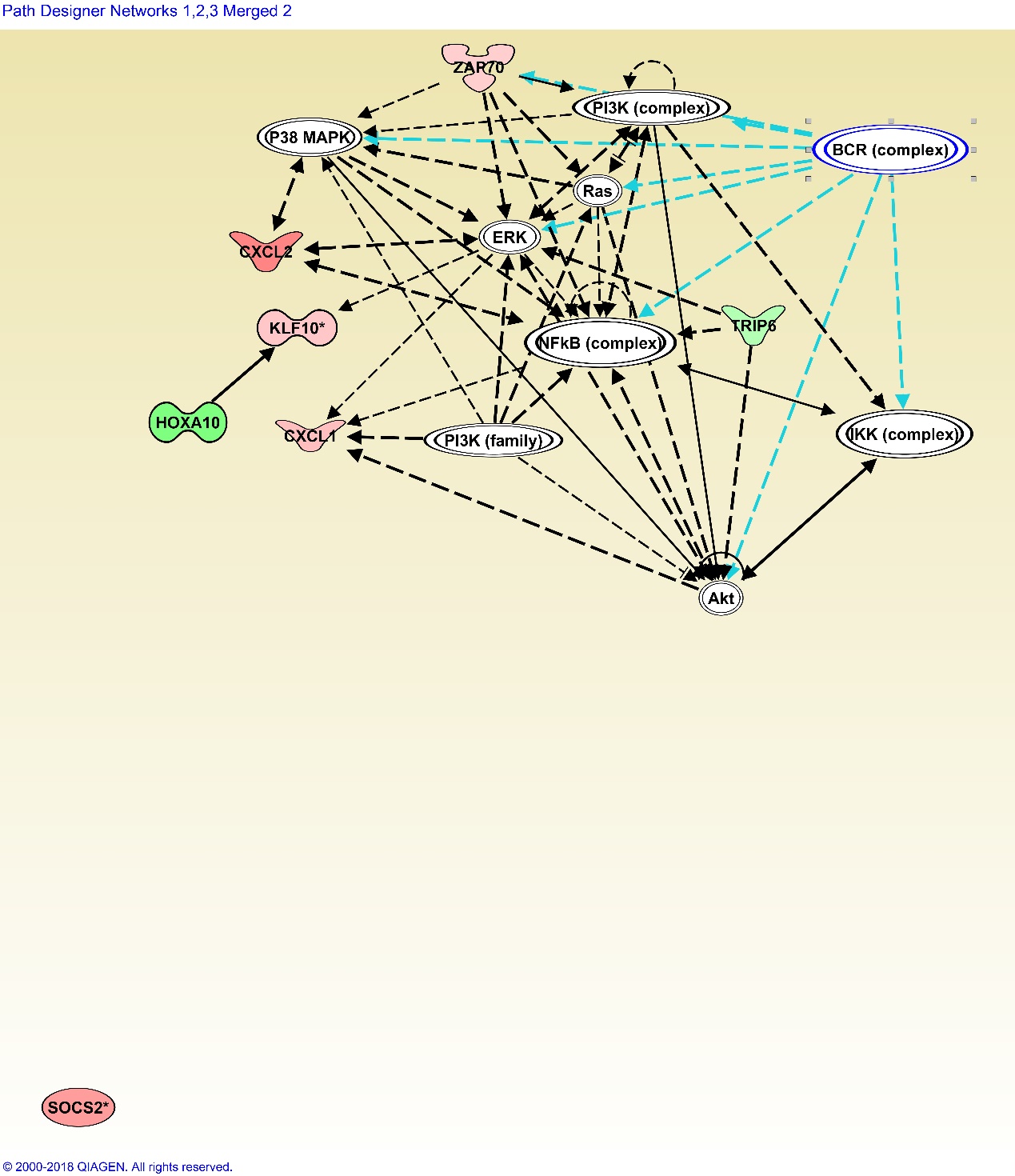
**

**Supplementary Figure 4**

**GSEA enrichment plots of gene sets in (a) *GATA2* ZF1-mutated group (b) *GATA2* ZF2-mutated group**

**(A)**


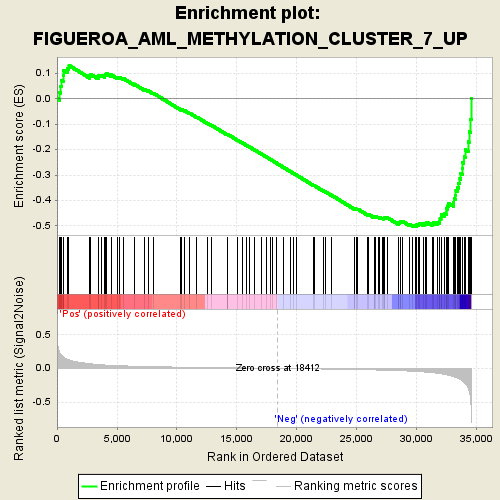


1. **ZF2**


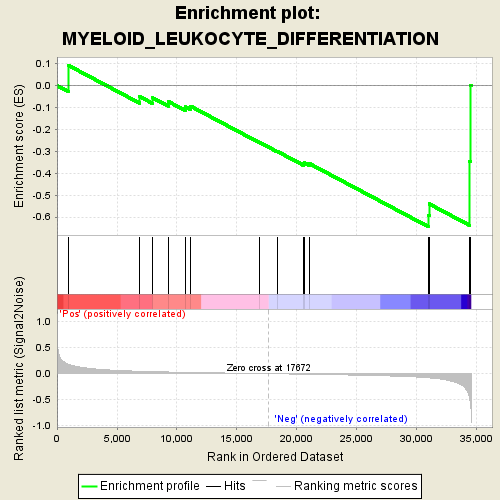


**Supplementary Table 1. Comparison of cytogenetic changes between AML patients with *GATA2* ZF1 and ZF2 mutations^a^**

| **Variables** | **Wild type**  **N=626 (%)** | ***GATA2* Mutated**  **N=43 (%)** | **ZF1 mutations**  **N=30 (%)** | **ZF2 mutations**  **N=10 (%)** | **P value^d^** | **P value**^e^ | **P value^f^** |
| --- | --- | --- | --- | --- | --- | --- | --- |
| **Karyotype**^b^ |  |  |  |  |  |  |  |
| Favorable | 85 (13.6) | 0 (0) | 0 (0) | 0 (0) | 0.0002 | 0.024 | 0.376 |
| Intermediate | 444 (70.9) | 42 (97.7) | 30 (100) | 10 (100) | <0.0001 | <0.0001 | 0.036 |
| Unfavorable | 97 (15.5) | 1 (2.3) | 0 (0) | 0 (0) | 0.026 | 0.014 | 0.372 |
| Normal | 291 (46.5) | 31 (72.1) | 22 (73.3) | 7 (70) | 0.001 | 0.004 | 0.202 |
| Complex | 76 (12.1) | 0 (0) | 0 (0) | 0 (0) | 0.010 | 0.038 | 0.617 |
| t(8;21) | 57 (9.1) | 0 (0) | 0 (0) | 0 (0) | 0.042 | 0.099 | >0.999 |
| Inv(16) | 26 (4.2) | 0 (2.3) | 0 (0) | 0 (0) | 0.402 | 0.625 | >0.999 |
| -5/5q^c^ | 34 (5.4) | 0 (0) | 0 (0) | 0 (0) | 0.158 | 0.394 | >0.999 |
| -7/7q^c^ | 43 (6.9) | 1 (2.3) | 0 (0) | 0 (0) | 0.350 | 0.251 | >0.999 |
| -20/20q | 6 (1.0) | 0 (0) | 0 (0) | 0 (0) | >0.999 | >0.999 | >0.999 |
| +8^c^ | 54 (8.6) | 2 (4.5) | 2 (6.7) | 0 (0) | 0.568 | >0.999 | >0.999 |
| +11^c^ | 14 (2.2) | 0 (0) | 0 (0) | 0 (0) | >0.999 | >0.999 | >0.999 |
| +13^c^ | 7 (1.1) | 0 (0) | 0 (0) | 0 (0) | >0.999 | >0.999 | >0.999 |
| +21^c^ | 22 (3.5) | 2 (4.5) | 2 (6.7) | 0 (0) | 0.662 | 0.301 | >0.999 |
| t(7;11) | 8 (1.3) | 1 (2.3) | 0 (0) | 1 (10) | 0.452 | >0.999 | 0.134 |
| t(3;3) | 6 (1.0) | 2 (4.5) | 2 (6.7) | 0 (0) | 0.088 | 0.048 | >0.999 |

^a^Cytogenetic data were available in 669 patients among total cohort including 43 patients with GATA2 mutations and 626 without the mutations. Among 43 patients with *GATA2* mutations, 3 patients with mutations outside the ZF domains were not included.

^b^Favorable, t(8;21), inv (16) ; unfavorable, -7, del(7q), -5, del(5q), 3q abnormality, complex abnormalities; Intermediate, normal karyotype and other abnormalities.

^c^Only including simple chromosomal abnormalities with 2 or less changes, but not those with complex abnormalities with 3 or more aberrations.

^d^*GATA2*-mutated patients vs *GATA2* wild-type patients

^e^*GATA2* ZF1-mutated patients vs *GATA2* wild-type patients

^f^*GATA2* ZF2-mutated patients vs *GATA2* wild-type patients

**Supplementary Table 2. Multivariate analysis (Cox regression) on the DFS and OS in total 469 AML patients who received standard intensive chemotherapy**

|  | **OS** | | | **DFS** | | |
| --- | --- | --- | --- | --- | --- | --- |
| **Variables** | **RR** | **95% CI** | **P value** | **RR** | **95% CI** | **P value** |
| Age^a^ | 2.911 | 2.110-4.015 | <0.0001 | 1.588 | 1.250-2.017 | <0.0001 |
| WBC^b^ | 2.176 | 1.577-3.001 | <0.0001 | 1.733 | 1.349-2.228 | <0.0001 |
| Karyotype^c^ | 3.650 | 2.415-5.517 | <0.0001 | 2.208 | 1.538-3.169 | <0.0001 |
| *NPM1*/*FLT3*-ITD^d^ | 0.429 | 0.246-0.749 | 0.003 | .428 | 0.269-0.679 | <0.0001 |
| *CEBPA*^dm^ | 0.542 | 0.267-1.100 | 0.090 | .568 | 0.354-0.911 | 0.019 |
| *GATA2* ZF1 mutation | 0.323 | 0.093-1.125 | 0.076 | .790 | 0.405-1.542 | 0.490 |

Abbreviations: RR, relative risk; CI, confidence interval,

^a^Age> 50 relative to Age ≤50 (the reference)

^b^WBC greater than 50,000/μL vs. 50,000/μL or less

^c^*NPM1*^mut^/*FLT3-*ITD^neg^ vs. other subtypes

^d^Unfavorable cytogenetics vs. others

**Supplementary Table 3. Distinct characteristics associated with *GATA2* ZF1 and ZF2 mutations**

|  | ***GATA2* ZF1 mutations** | ***GATA2* ZF2 mutations** |
| --- | --- | --- |
| **Clinical Features*** | Median age, 39  Mostly FAB M1 | Medial age, 47  Mostly FAB M2 |
| **Cytogenetics*** | All intermediate-risk | All intermediate-risk |
| **Mutations*** | Fewer *NPM1*, *FLT3*/ITD  Strong *CEBPA*^dm^ association | Higher *NPM1*, *FLT3*/ITD  Weak *CEBPA*^dm^ association |
| **Clinical outcome*** | Higher CR rate  Better OS, DFS | No survival benefit |
| **Disease spectrum**** | Familial AML ^11^  Childhoold MDS ^5^  *De novo* AML | Familial MDS/ AML ^4^  MonoMAC syndrome ^8^  CML blast crisis ^3^  Childhoold MDS ^5^ |

***** from our study

**** f**rom literature

Abbreviations: *CEBPA*^dm^*, CEBPA^double-mutation^;* MonoMAC, Monocytopenia and mycobacterial infection
